# Supplementary material for: Sacral-Nerve-Sparing Planning Strategy in Pelvic Sarcomas/Chordomas Treated with Carbon-Ion Radiotherapy
Source: Cancers (Basel). 2024 Mar 26;16(7):1284. doi: 10.3390/cancers16071284 (PMC11010899; doi:10.3390/cancers16071284)
Supplement: Supplementary file 1 [file cancers-16-01284-s001.zip › Supplementary Figures S1-S5.pdf]

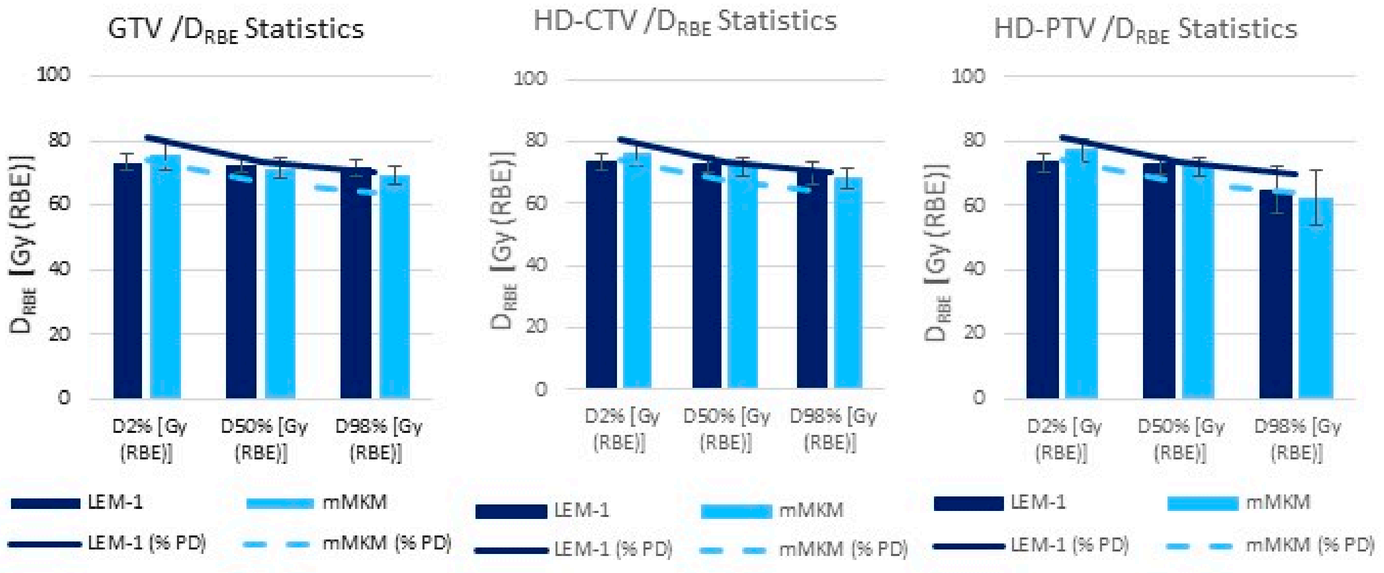

Figure S1. $D_{RBE|LEM-I}$ and $D_{RBE|mMKM}$ statistics for GTV, HD-CTV and HD-PTV : D2%, D50%, D98%. Solid and dashed line represent threshold for respective clinical goal.

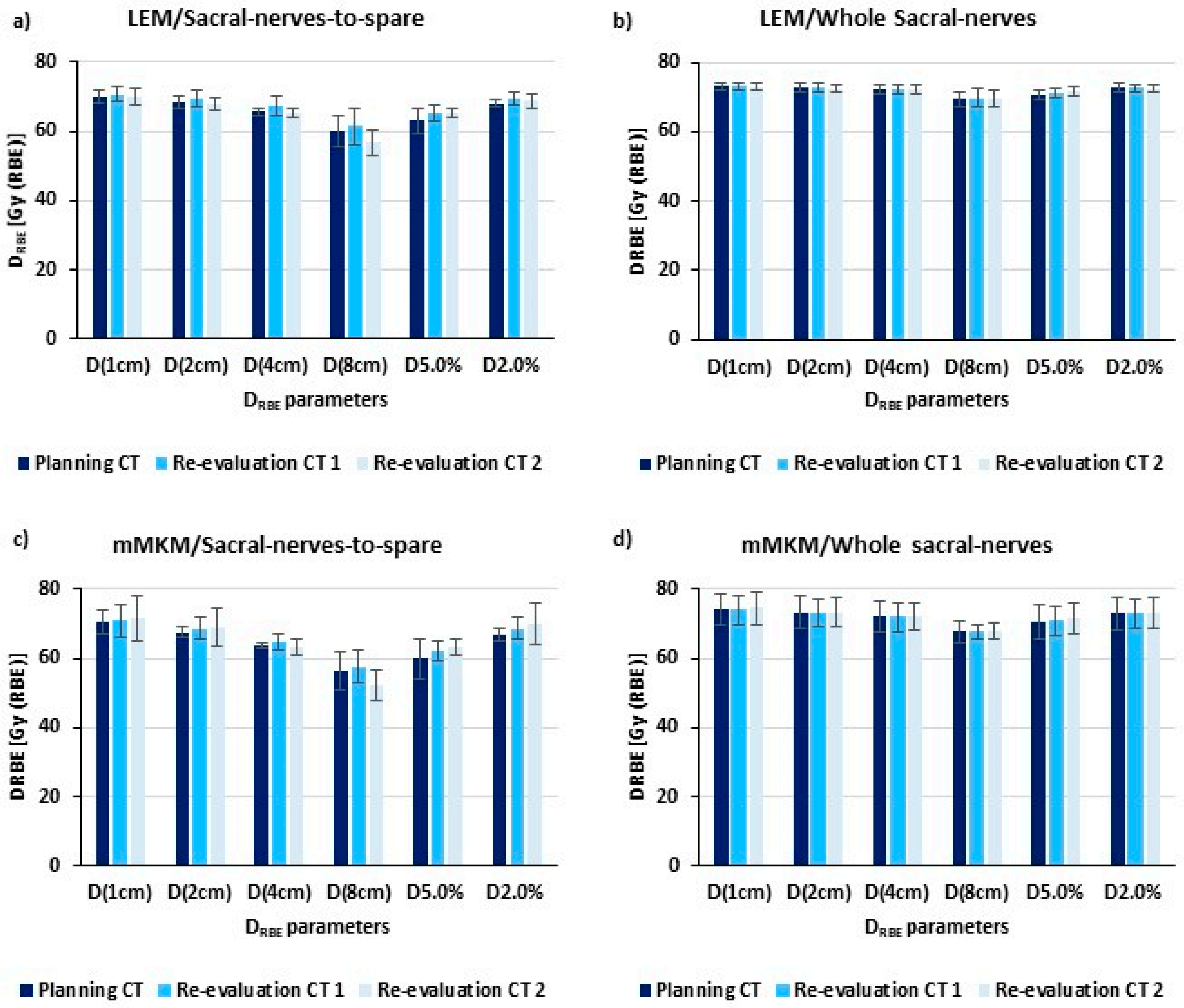

Figure S2. Robustness analysis SNSo-CIRT strategy: Comparison of $D_{RBE|LEM-I}$ and $D_{RBE|mMKM}$ statistics for sacral-nerves-to-spare and whole sacral-nerves on re-evaluation CT scans compared to original planning CT. (n = 7).

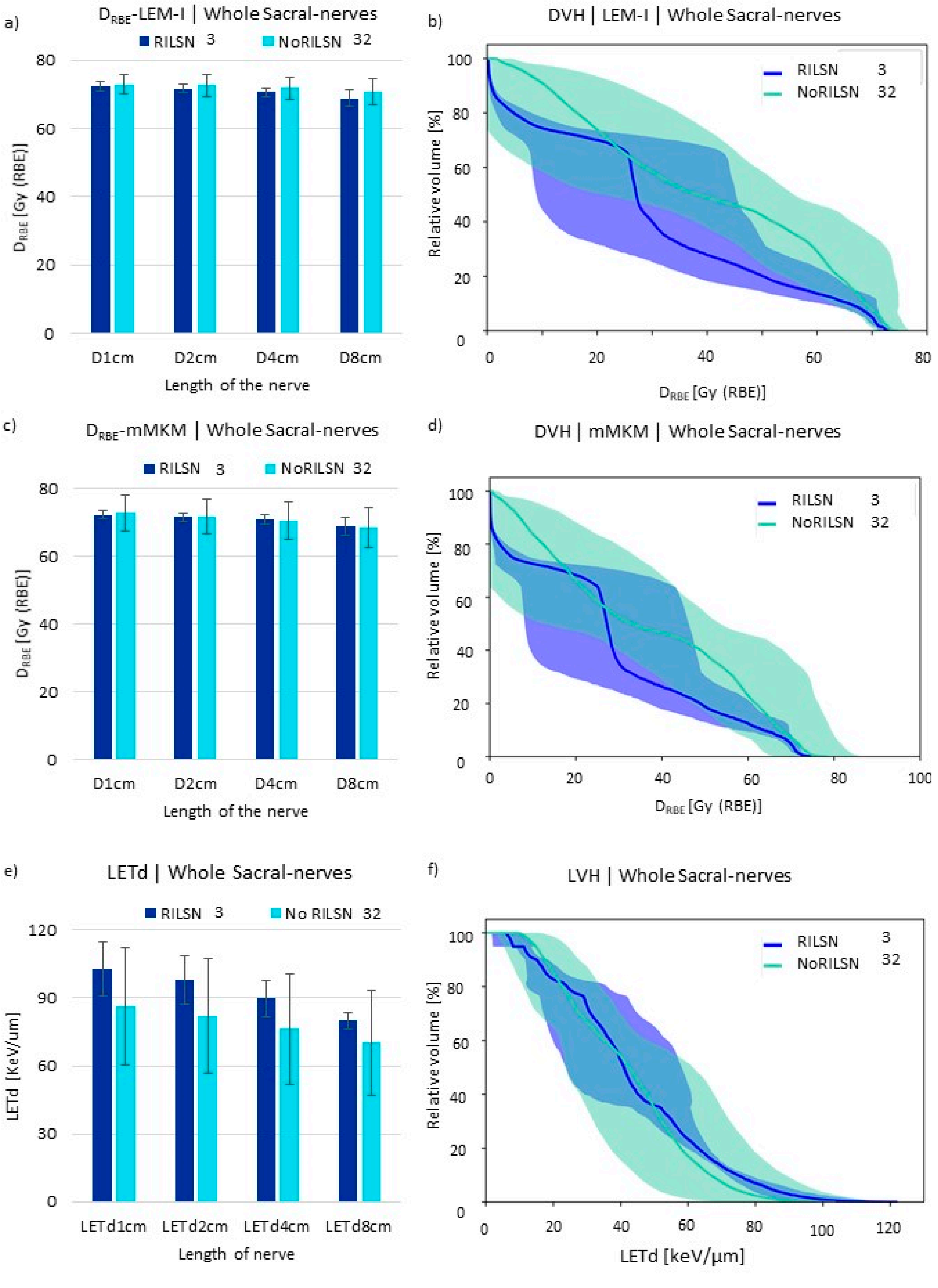

Figure S3. $D_{RBE|LEM-I}$ and $D_{RBE|mMKM}$ and LETd statistics. a) LEM-I doses, c) mMKM doses and e) LETd received by 1 cm, 2 cm, 4 cm and 8 cm lengths of the whole sacral nerves in patients with and without RILSN. DVH analysis of whole sacral nerves in patients with and without RILSN with respect to b) LEM-I and d) mMKM model-based dose calculation. f) Relative- volume LVH of the. whole sacral nerves of patient with RILSN and those without RILSN.

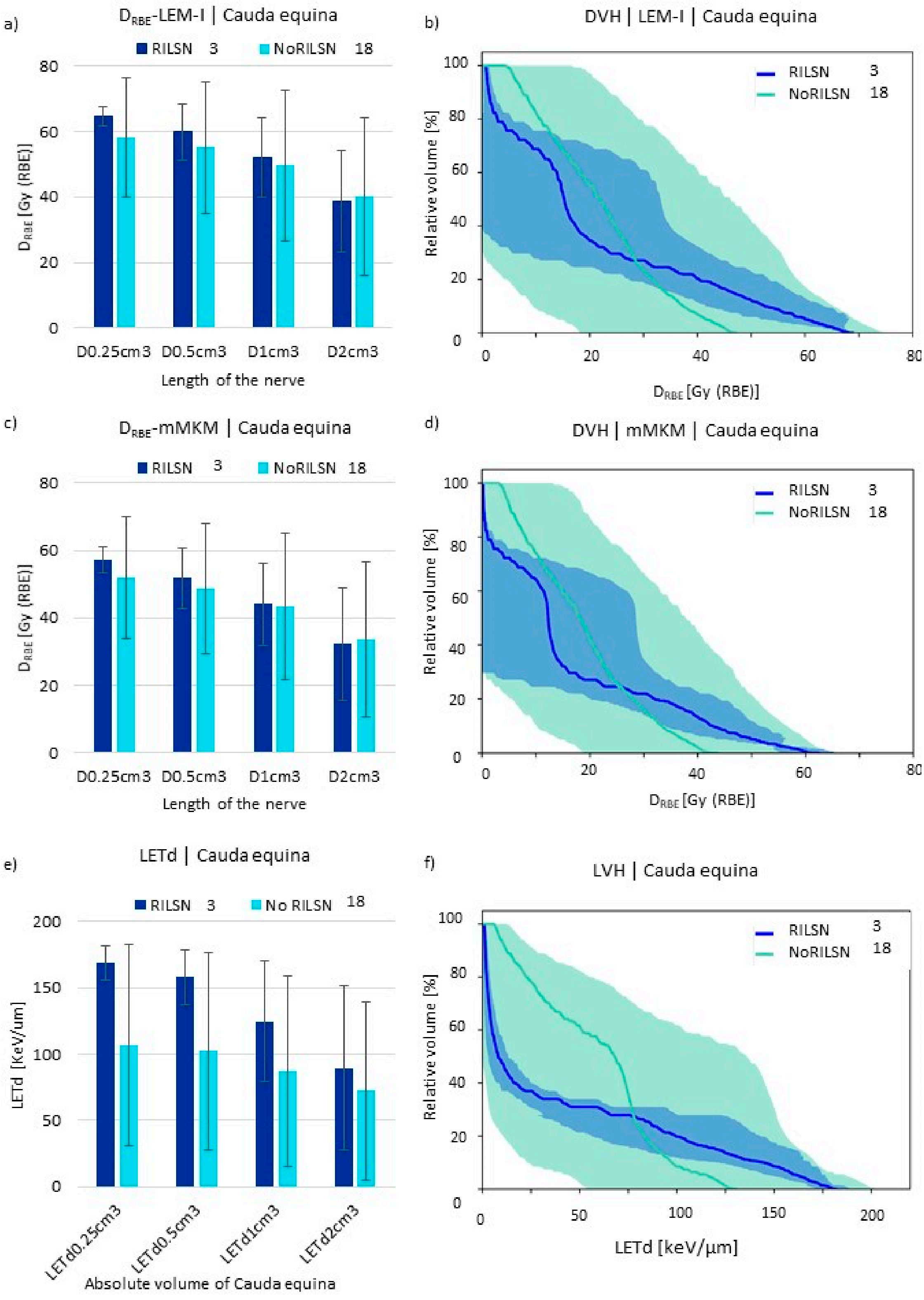

Figure S4. $D_{RBE|LEM\text{-}I}$ and $D_{RBE|mMKM}$ and LETd statistics. a) LEM-I doses, c) mMKM doses and e) LETd received by doses to 0.25 cm^3, 0.5 cm^3, 1 cm^3, 2 cm^3 of cauda equina in patients with and without RILSN. DVH analysis of cauda equina in patients with and without RILSN with respect to b) LEM-I and d) mMKM model-based dose calculation. f) Relative- volume LVH of the. cauda equina of patient with RILSN and those without RILSN. (p = not significant).

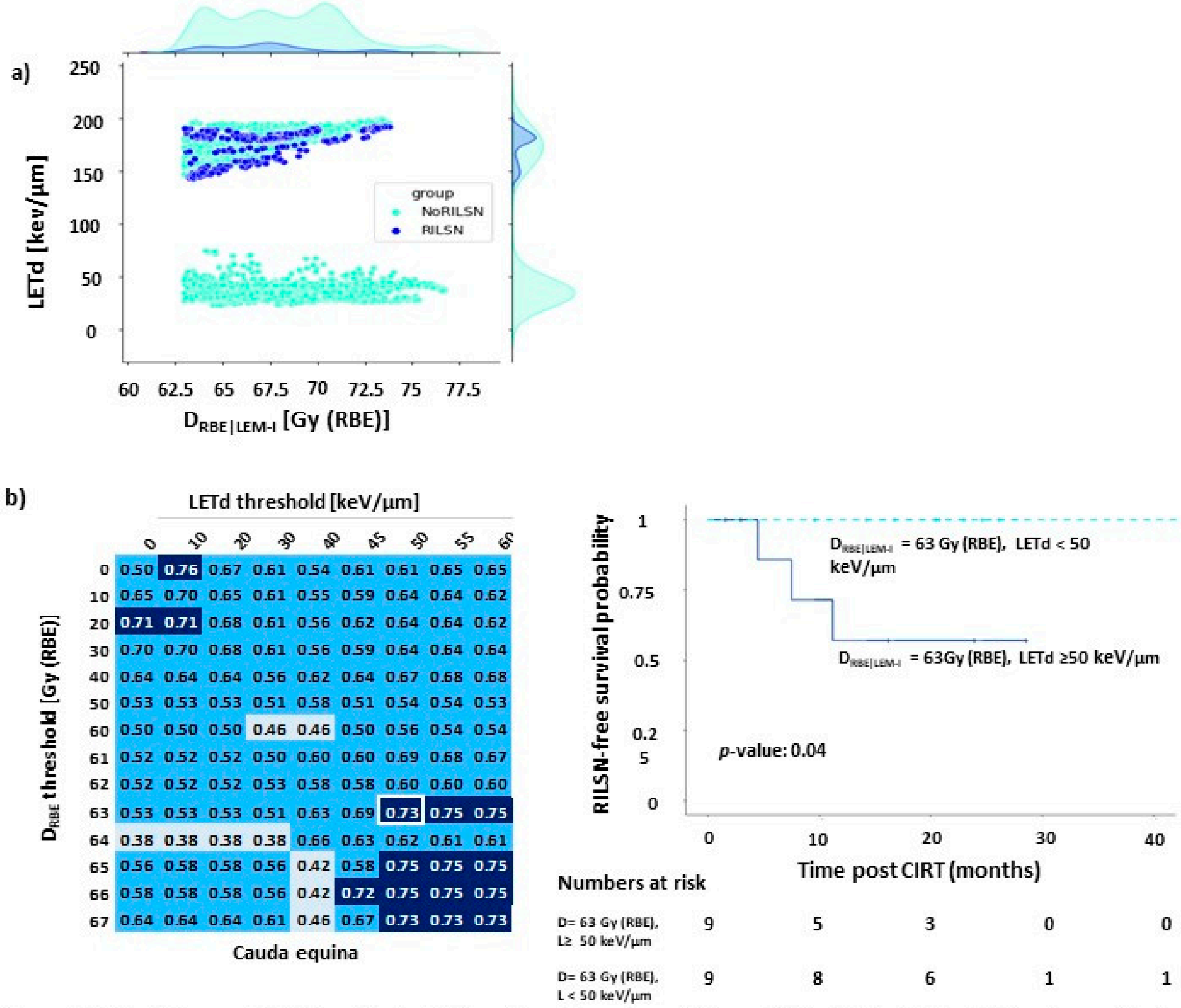

Figure S5: Voxel by voxel LETd analysis plot for a) cauda equina at D_{RBE} cutoff = 63 Gy (RBE). AUC value matrix for D_{RBE}-filtered-LETd for b) cauda equina. c) Kaplan Meier survival analysis of cases with for $D_{RBE|LEM-I}$ cutoff = 63 Gy (RBE) and LETd cutoff = 50 keV/and µm for (Note: n: RILSN = 3, NoRILSN = 15).
