# Supplementary material for: Sacral-Nerve-Sparing Planning Strategy in Pelvic Sarcomas/Chordomas Treated with Carbon-Ion Radiotherapy
Source: Cancers (Basel). 2024 Mar 26;16(7):1284. doi: 10.3390/cancers16071284 (PMC11010899; doi:10.3390/cancers16071284)
Supplement: Supplementary file 1 [file cancers-16-01284-s001.zip › Supplementary Methods.pdf]

Supplementary Methods:

To assess optimal D_{RBE} and LETd threshold we evaluated all the thresholds with corresponding AUC values > 0.7 and then selected one with highest safe D_{RBE} and LETd (Figure 4). RILSN-free survival at $D_{RBE|LEM-I}$ cutoff = 64 Gy (RBE), in patients with < 12% voxels of sacral-nerves-to-spare receiving LETd < 55 keV/μm had significantly higher 2-year RILSN-free survival (100%) than those with LETd ⩾ 55 keV/μm (75%) (p< 0.05). The sensitivity at $D_{RBE|LEM-I}$ cutoff = 64 Gy (RBE), and LETd = 55 keV/μm was 100% and specificity was 67%. Hence, $D_{RBE|LEM-I}$ cutoff = 64 Gy (RBE), and LETd = 55 keV/μm was considered appropriate predictor. Appropriate thresholds for whole sacral-nerves and cauda equina were selected following the same approach.
