# Supplementary material for: Sacral-Nerve-Sparing Planning Strategy in Pelvic Sarcomas/Chordomas Treated with Carbon-Ion Radiotherapy
Source: Cancers (Basel). 2024 Mar 26;16(7):1284. doi: 10.3390/cancers16071284 (PMC11010899; doi:10.3390/cancers16071284)
Supplement: Supplementary file 1 [file cancers-16-01284-s001.zip › Supplementary Table S1.pdf]

Supplementary Table S1: Abbreviation list:

CIRT	Carbon-ion therapy
SNSo-CIRT	Sacral nerve sparing optimized carbon-ion therapy
RBE	Relative Biological effectiveness
LEM-I	Local effect model-I
mMKM	Modified microdosimetric kinetic model
MBM	Mixed beam model
LETd	D/ose-averaged linear energy transfer
HD-CTV	High dose clinical target volume
$D_{RBE\|LEM\text{-}I}$, $D_{RBE\|mMKM}$	RBE-weighted dose (calculated by LEM-I /mMKM model)
$D_{RBE\|LEM\text{-}I\|'x'\%}$ or $D_{'x'\%}$	RBE weighted dose received by 'x'% target/OAR volume
$D_{x'cm}$	RBE weighted dose received by 'x' cm length of nerve
$LETd_{'x'\%}$	LETd received by 'x'% target/OAR volume
$LETd_{'x'cm}$	LETd received by 'x' cm length of nerve
D_{RBE}-filtered-LETd	LETd in volume receiving > D_{RBE} cutoff/threshold
